# Multidrug resistant *Pseudomonas aeruginosa* in Estonian hospitals

**DOI:** 10.1186/s12879-018-3421-1

**Published:** 2018-10-11

**Authors:** Kaidi Telling, Mailis Laht, Age Brauer, Maido Remm, Veljo Kisand, Matti Maimets, Tanel Tenson, Irja Lutsar

**Affiliations:** 1 Department of Microbiology, Institute of Biomedicine and Translational Medicine, University of Tartu, Ravila 19, 50411 Tartu, Estonia; 2 Institute of Technology, University of Tartu, Tartu, Estonia; 3 Institute of Molecular and Cell Biology, University of Tartu, Tartu, Estonia; 4 Department of Infection Control, Tartu University Hospital, Tartu, Estonia

**Keywords:** Carbapenem resistance, Outbreak, WGS (whole-genome sequencing), Beta-lactamases

## Abstract

**Background:**

We aimed to identify the main spreading clones, describe the resistance mechanisms associated with carbapenem- and/or multidrug-resistant *P. aeruginosa* and characterize patients at risk of acquiring these strains in Estonian hospitals.

**Methods:**

Ninety-two non-duplicated carbapenem- and/or multidrug-resistant *P. aeruginosa* strains were collected between 27th March 2012 and 30th April 2013. Clinical data of the patients was obtained retrospectively from the medical charts. Clonal relationships of the strains were determined by whole genome sequencing and analyzed by multi-locus sequence typing. The presence of resistance genes and beta-lactamases and their origin was determined. Combined-disk method and PCR was used to evaluate carbapenemase and metallo-beta-lactamase production.

**Results:**

Forty-three strains were carbapenem-resistant, 11 were multidrug-resistant and 38 were both carbapenem- and multidrug-resistant. Most strains (54%) were isolated from respiratory secretions and caused an infection (74%). Over half of the patients (57%) were ≥ 65 years old and 85% had ≥1 co-morbidity; 96% had contacts with healthcare and/or had received antimicrobial treatment in the previous 90 days.

Clinically relevant beta-lactamases (OXA-101, OXA-2 and GES-5) were found in 12% of strains, 27% of which were located in plasmids. No Ambler class B beta-lactamases were detected. Aminoglycoside modifying enzymes were found in 15% of the strains. *OprD* was defective in 13% of the strains (all with CR phenotype); carbapenem resistance triggering mutations (F170 L, W277X, S403P) were present in 29% of the strains. Ciprofloxacin resistance correlated well with mutations in topoisomerase genes gyrA (T83I, D87N) and parC (S87 L). Almost all strains (97%) with these mutations showed ciprofloxacin-resistant phenotype.

Multi-locus sequence type analysis indicated high diversity at the strain level – 36 different sequence types being detected. Two sequence types (ST108 (*n* = 23) and ST260 (*n* = 18)) predominated. Whereas ST108 was associated with localized spread in one hospital and mostly carbapenem-resistant phenotype, ST260 strains occurred in all hospitals, mostly with multi-resistant phenotype and carried different resistance genotype/machinery.

**Conclusions:**

Diverse spread of local rather than international *P. aeruginosa* strains harboring multiple chromosomal mutations, but not plasmid-mediated Ambler class B beta-lactamases, were found in Estonian hospitals.

**Trial registration:**

This trial was registered retrospectively in ClinicalTrials.gov (NCT03343119).

**Electronic supplementary material:**

The online version of this article (10.1186/s12879-018-3421-1) contains supplementary material, which is available to authorized users.

## Background

*Pseudomonas aeruginosa* (PA) is an opportunistic pathogen present in many ecological settings. It can survive in living (humans, animals, plants) and non-living (water, soil, artificial surfaces) sources [1], but is rarely found in the microbiota of healthy humans [2]. The colonization rate by PA significantly increases (reaching up to 80%) in patients with chronic illnesses (e.g. cystic fibrosis, severe burns) or extensive exposure to healthcare facilities involving interruption of protective barriers [3–5].

PA is exceptionally flexible, using different regulatory and metabolic mechanisms to adapt to antibiotic pressure. It has an intrinsic resistance to wide range of antimicrobial agents, and a high capacity to attain resistance mutations and mobile genetic elements [6].

According to the European Centre for Disease Prevention and Control (ECDC) in 2016, resistance of PA in most European countries exceeded 10% of all antimicrobials investigated. Furthermore, the prevalence of MDR-PA is rising globally, a phenomenon mainly associated with the spread of high-risk clones (e.g. multi-locus sequence types ST235, ST111, ST175) associated with nosocomial outbreaks and transferable resistance mechanisms, especially horizontally-acquired beta-lactamases [7]. Despite the low antibiotic consumption comparable to other North European countries [8], the resistance rates of PA in Estonia, especially to carbapenems, are much higher than in other low-end usage countries, with the trends becoming alarming. In 2012, 12.5% of the strains reported to the ECDC were carbapenem-resistant (CR), but this had risen above 20% by 2016 [9, 10].

Although main trigger associated with CR in PA is production of plasmid-mediated beta-lactamases/carbapenemases, mutational resistance mechanisms in chromosomal genes – e.g. altered expression of outer membrane porins or efflux systems and increased chromosomal cephalosporinase (AmpC) activity, may all have affected the development of resistance [7].

Unfortunately data provided by the ECDC reflects just a fraction of the actual situation, as only invasive strains and the phenotypic resistance are reported. This leaves a gap in terms of non-invasive infections, and in genetic information of the spreading bacterial lineages and resistance machinery they carry. Previous studies conducted in Estonia are no exception; they have included only certain patient groups (intensive care units) [11] or blood-stream infections [12] without any information at the molecular level. Both knowledge of main risk groups and genetic data of strains is essential in understanding resistance transfer, and to take actions needed to stop it.

## Methods

We aimed to characterize hospitalized patients carrying carbapenem- or/and multidrug- resistant *P. aeruginosa* (CR/MDR-PA), identifying the main spreading clones and describing the most important resistance mechanisms, including the occurrence of clinically relevant beta-lactamases.

### Study design and settings

The study was conducted in 5 major Estonian hospitals treating both pediatric and adult patients - 2 3rd level multidisciplinary referral hospitals (Tartu University Hospital and North Estonia Medical Centre), 2 central hospitals (West Tallinn Central Hospital and East-Tallinn Central Hospital) and 1 private hospital specializing in plastic and vascular surgery (The Hospital of Reconstructive Surgery).

During the study period all consecutive PA strains isolated from hospitalized patients taken by discretion of treating physicians on suspicion or confirmed infection and identified as CR and/or MDR by the microbiology laboratories of the hospitals were collected, with a target of 100 strains. Only one strain was included from each patient (invasive strain or firstly isolated strain). Strains from ambulatory patients and clinics were excluded. The collection lasted from 27th of March 2012 to 30th of April 2013.

### Clinical data collection

Hospital records of patients with eligible PA strains were reviewed retrospectively to obtain demographic data (age, gender), the presence of major co-morbidities and predisposing clinical conditions, date and reason of admission, site of infection, in-hospital movement, presence of infection or colonization caused by CR/MDR-PA, and the outcome*.*

Specifically, we recorded the presence of invasive devices, surgery within the previous 30 days, hospitalization, or time spent in a long-term care facility, and antimicrobial therapy in the previous 90 days.

### Sampling and microbiological methods

Standard clinical laboratory methods were used to isolate and identify PA from clinical specimens*.* Briefly, urine was plated on cysteine lactose electrolyte-deficient agar, respiratory samples on blood and chocolate agar, which were incubated at 37 °C for 24 to 48 h. Tissue biopsies were homogenized and incubated in thioglycolate broth at 37 °C for 14 days. Blood, cerebrospinal, pleural and abdominal fluids were processed and monitored with a BACTEC 9240 blood culture system (Becton Dickinson, Sparks, MD, USA). One colony with *Pseudomonas*-like morphology was identified using classical biochemical tests (catalase and oxidase reactions) and VITEK2 Compact or API tests (bioMérieux, Marcy l’Etoile, France).

Finally, all PA isolates were confirmed by using matrix-assisted laser desorption ionization time-of-flight mass spectrometry (Bruker Daltonics, Bremen, Germany). Only one isolate per patient was included in the final analysis, giving preference to more invasive strains (blood or cerebrospinal fluid).

### Phenotypic susceptibility testing

The MIC for 9 antipseudomonal antibiotics (ceftazidime, cefepime, meropenem, imipenem, piperacillin/tazobactam, amikacin, gentamicin, tobramycin and ciprofloxacin) for each strain was measured with an epsilometer (E-test, bioMérieux, Marcy l’Etoile, France), with the quality control strain ATCC® 27,853™ being routinely used. Antibiotic susceptibility was determined using EUCAST breakpoints and definitions [13, 14]. Strains non-susceptible to at least one tested carbapenem were designated CR, whereas those not susceptible to 3 or more antibiotic classes were defined as MDR [13].

Forty-seven CR strains were screened for *Klebsiella pneumoniae* carbapenemase (KPC) and metallo-beta-lactamase (MBL) production by combined-disk method using disks containing 10 μg imipenem, 10 μg imipenem with phenylboronic acid, 10 μg imipenem with cloxacillin high, 10 μg imipenem with dipicolinic acid and imipenem with EDTA (Rosco Diagnostica, Taastrup, Denmark). Quality control strains were *P. aeruginosa* CCUG59626, *K. pneumoniae* BAA1705 and *E. coli* ATCC® 25,922™.

PCR-based test for detection of genes encoding carbapenemases was used according to Poirel et al. [15] on the same 47 strains for double-control. Three different multiplex reaction mixtures were defined and evaluated for the detection of MBL-encoding genes (***bla***_IMP_, ***bla***_VIM,_ ***bla***_GIM_ and ***bla***_NDM_), class A carbapenemase gene ***bla***_KPC_ and class D carbapenemase gene ***bla***_oxa-48._ The following control strains were used - Swedish Institute for Communicable Disease Control (Sweden) carbapenemases detection control set: OXA-48-positive *K. pneumoniae* Oxa241, KPC-positive *K. pneumoniae* K271, NDM-1-positive *K. pneumoniae* K275, IMP-positive *P. aeruginosa* CCUG59626, VIM-positive *K. pneumoniae* CCUG58547, GIM-positive *P. aeruginosa;* and KPC-positive *K. pneumoniae* BAA1705.

### DNA extraction

A modified GuSCN-silica protocol was used for the DNA extraction from a single colony [16]. Briefly, cells were transferred into a solution containing 570 μl TE (pH 7.6) buffer (TRIS-EDTA) and 30 μL 10% SDS, with ~ 0.5 g zirconium beads (0.1 mm diameter), which was processed by 5 min on bead beater (Minibead beater, Bio-Spec Products, Bartlesville, USA), followed by centrifugation at 10000 rpm for 1 min. Cells were lysed by combining the supernatant with lysis buffer L6 (5.25 M GuSCN, 100 mM Tris – HCl, pH 6.4, 20 mM EDTA, 1.3% Triton X-100). Custom-prepared silica suspension (40 μl) was added before incubation for 5 min at room temperature and centrifugation at 5000 rpm for 10 s. The supernatant was discarded and the pellet washed with buffer L2 (5 M GuSCN) and 50% ethanol. The silica pellet was briefly dried and the DNA eluted in ultra-pure water (milli-Q). The extracted DNA was stored at − 20 °C until analyzed.

### Whole genome sequencing

Total bacterial DNA was quantified using a Qubit® 2.0 Fluorometer (Invitrogen, Grand Island, USA) and 2200 TapeStation (Agilent Technologies, Santa Clara, USA). Ten nanograms of sample DNA was processed using an Illumina Nextera XT sample preparation kit (Illumina, San Diego, USA). The resulting DNA libraries were validated by qPCR using a Kapa Library Quantification Kit (Kapa Biosystems, Woburn, USA) to optimize cluster generation.

Ninety-two ssDNA Nextera XT libraries originating from 92 different clones were pooled and sequenced in one rapid-output run of HiSeq2500 (Illumina, San Diego, USA), with paired-end, 150-bp reads. Demultiplexing was done with CASAVA 1.8.2. (Illumina, San Diego, USA), allowing one mismatch in the index reads.

### Draft assembly of whole genome sequences (WGS), multi-locus sequence typing (MLST) and phylogeny analysis

All Illumina reads were assembled de novo using the SPAdes genome assembler (ver 3.5.0), together with the MismatchCorrector [17].

A BLAST-based tool from https://cge.cbs.dtu.dk/services/MLST/ was run to annotate the MLST fragments within the WGS data [18]. To identify the sequence types (ST), the batch profile query from the pubMLST website for PA (http://pubmlst.org/paeruginosa) together with their locus/sequence definitions was used.

Sequences were aligned using global genome alignment to determine the core genomes. Thereafter, recombinations in the core genomes were detected using BratNextGen software [19]. For phylogenetic analysis, recombination-free alignments were created by masking all significant recombinant segments as missing data in the input alignment. These alignments were used to reconstruct a maximum likelihood phylogenetic tree with RaxML, using the GTR-GAMMA model.

As core genome alignment and MLST analysis resulted in similar clustering, the data are presented according to STs of MLST.

### Identifying resistance genes

Antibiotic resistance genes were identified using a homology search against the collection of antibiotic resistance protein sequences from The Comprehensive Antibiotic Resistance Database (CARD, http://arpcard.mcmaster.ca/, version 1.1.0) and beta-lactamases from http://www.lahey.org/studies/. *GyrA, ParC, OprD, Rmt and Arm* sequences were retrieved from the NCBI protein database. Identity and coverage thresholds were set to 90%.

Clinical relevance of found beta-lactamases was assessed as suggested by Potron et al. [20]

The chromosomal or plasmid origin of beta-lactamase genes was determined using a blastn search of corresponding contigs against the NCBI nt/nr database. Top matches were also examined manually to determine whether they were plasmid or chromosomal sequences. We could not decide whether the beta-lactamase gene was located in plasmid or chromosome for very short contigs and/or contigs with low coverage matches, or matches against both chromosomal and plasmid sequences. Contigs matching only against chromosomal genome sequences were not considered to be plasmid-related, whereas contigs matching with high coverage (> 95%) and identity (> 98%) to only plasmid sequences were considered as possibly originating from the plasmids.

### Definitions and statistical analysis

For the analysis of demographic and clinical characteristics, patients were categorized as infected and colonized according to following criteria. Colonization was defined as absence of clinical signs of infection on day of isolation PA in the anatomical site were microorganism was detected. Infections were classified by their most probable origin of acquisition to community and hospital-acquired. ECDC definitions for hospital-acquired infections were used [21]. The Charlson weighted index of comorbidity was calculated using an updated (ICD-10 diagnosis-based) version [22].

Descriptive analysis used R 2.8.1 [23]. Chi-square or Fisher’s exact tests were run where appropriate for categorical and the Kolmogorov-Smirnov test for continuous variables. Adjustment for multiple testing was made using the Bonferroni method.

## Results

The local microbiology laboratories identified a total of 118 CR/MDR-PA strains, of which 26 were excluded from the final analysis (16 from ambulatory patients, 6 were duplicates from the same patient, and 4 were not PA according to MALDI-TOF). Of the 92 strains, 43 were CR, 11 were MDR, and 38 were both MDR and CR.

The most frequently represented sources were respiratory secretions (*n* = 50; 54%), followed by wound aspirates (*n* = 22; 24%), urine (*n* = 12; 13%) and materials retrieved during intra-abdominal or vaginal procedures or surgeries (*n* = 7; 8%). One strain originated from the bloodstream.

The median time elapsed between hospital admission and CR/MDR-PA isolation was 13 days (IQR: 6–27 days).

### Study population and characteristics

Sixty-eight (74%) patients had infections, whereas 24 (26%) were classified as asymptomatic carriers. The most common infections were pneumonia (*n* = 34; 50%), skin and soft tissue (*n* = 14; 21%), surgical site (*n* = 6; 9%), and intra-abdominal infections (n = 5; 7%). About half the patients (*n* = 44; 47%) had hospital-acquired infections (Additional file 1: Table S1).

The demographic and clinical characteristics of patients that had CR/MDR-PA are presented in Table 1.

**Table 1:** The most important clinical characteristics of patients with CR/MDR-PA infection or colonization

	All (*n* = 92)	Infection (*n* = 68)	Colonization (*n* = 24)	OR (95% CI)
Demographic characteristics				
Male sex (%)	56 (61)	44 (65)	12 (52)	1.8 (0.7–4.7)
Age: median; years (IQR)	68 (52–74)	69 (51–74)	66 (52–75)	
Patients ≥65 years (%)	52 (57)	37 (54)	15 (63)	0.7 (0.3–1.9)
Previous contact with health care system				
Hospitalization within 90 days (%)	35 (38)	25 (37)	10 (42)	0.8 (0.3–2.1)
Long-term healthcare facilities stay within 90 days (%)	5 (5)	4 (6)	1 (4)	1.4 (0.2–13.5)
Antibiotic therapy in previous 90 days (%)	82 (89)	60 (88)	22 (92)	0.7 (0.1–3.5)
Surgery in previous 30 days (%)	53 (58)	38 (56)	15 (63)	0.8 (0.3–2.0)
Intensive care unit stay; duration in days: median (IQR)	57 (62); 15 (8–24.5)	40 (59)	17 (71)	0.6 (0.2–1.6)
Invasive procedures in previous 30 days				
Bronchoscopy	30 (33)	25 (37)	5 (21)	2.2 (0.7–6.7)
Hemodialysis	17 (19)	13 (19)	4 (17)	1.2 (0.3–4.1)
Endoscopy	17 (18)	14 (21)	3 (13)	1.8 (0.5–7.0)
Other	16 (17)	10 (15)	6 (25)	0.5 (0.2–1.6)
Invasive device use on the microorganism isolation day				
Mechanical ventilation of the lungs	47 (51)	34 (50)	13 (54)	0.8 (0.3–2.2)
Central venous catheter	48 (52)	33 (49)	15 (63)	0.6 (0.2–1.5)
Urinary catheter	51 (55)	38 (56)	13 (54)	1.1 (0.4–2.7)
Epicystostomy	9 (10)	7 (10)	2 (8)	1.3 (0.2–6.5)
Co-morbidities				
Congestive cardiac insufficiency	29 (32)	21 (31)	8 (33)	0.9 (0.3–2.4)
Chronic renal failure	21 (23)	16 (24)	5 (21)	1.2 (0.4–3.6)
Diabetes mellitus	20 (22)	15 (22)	5 (21)	1.1 (0.3–3.4)
Chronic pulmonary disease	17 (18)	12 (18)	5 (21)	0.8 (0.3–2.6)
Malignant tumor	16 (16)	9 (13)	7 (25)	0.4 (0.1–1.1)
Hemiplegia	14 (16)	11 (16)	3 (13)	1.4 (0.3–5.3)
Myocardial infarct	13 (14)	9 (13)	4 (17)	0.8 (0.2–2.8)
Cerebrovascular disease	12 (13)	11 (16)	1 (4)	4.4 (0.5–36.4)
Other^a^	34 (37)	27 (40)	7 (29)	1.6 (0.6–4.4)

^a^HIV, AIDS, hematologic malignancy, dementia, connective tissue disease, liver disease, peripheral vascular disease, ulcer disease, neutropenia, trauma, burn

Over half of the patients were elderly (≥65 years) with multiple co-morbidities (average (±SD), Charlson co-morbidity index (2.6 ± 2.1). About 85% patients had at least one co-morbidity. There were no patients < 18 years old.

Congestive cardiac insufficiency was commonly present (32%), followed by renal failure (23%) and diabetes mellitus (22%). Although 17 patients had chronic pulmonary disease, none had cystic fibrosis.

Most of the patients (*n* = 88; 96%) had preceding contact with the healthcare and/or received antibacterial treatment within the previous 90 days; beta-lactams were by far the most commonly used antibiotics (Additional file 2: Table S2).

There were no statistically significant differences detected in risk factors of colonized and infected patients probably due to small sample size and great variety in population analyzed. It might be assumed that factors affecting exposure to PA and leading from colonization to infection are quite diverse and need further research in more precisely selected groups to draw adequate conclusions.

Eighteen patients (20%) died during their hospital stay. The mortality rates in the 7 day, 30 day and 1 year after PA isolation were 2, 14 and 44%, respectively.

### Antibiotic susceptibility of PA

The MIC values together with the interpretations are shown in Table 2. The highest resistance rates were observed to imipenem and the lowest to amikacin (59.8 and 7.6%, respectively).

**Table 2:** Minimal inhibitory concentrations and antibiotic susceptibility interpretations of PA (*n* = 92)

Antibiotic	MIC range (mg/L)	MIC_50_ (mg/L)	MIC_90_ (mg/L)	S (%)	I (%)	R (%)
Beta-lactams						
Imipenem	0.5–32	24	32	19.6	20.7	59.8
Piperacillin/tazobactam	0.5–256	16	256	50.0	0	50.0
Meropenem	0.125–32	8	32	28.3	25.0	46.7
Cefepime	0.5–256	6	32	70.7	0	29.3
Ceftazidime	0.75–256	2	64	73.9	0	26.1
Ciprofloxacin	0.064–32	0.5	32	53.3	0	46.7
Aminoglycosides						
Gentamicin	0.125–256	2	48	80.4	0	19.6
Amikacin	1–256	6	16	75.0	17.4	7.6
Tobramycin	0.125–256	1	4	90.2	0	9.8

Abbreviations: *MIC*_*50*_ minimal inhibitory concentration inhibiting 50% of isolates, *MIC*_*90*_ minimal inhibitory concentration inhibiting 90% of isolates, *S* susceptible, *I* intermediately susceptible, *R* resistant

**Fig. 1:**
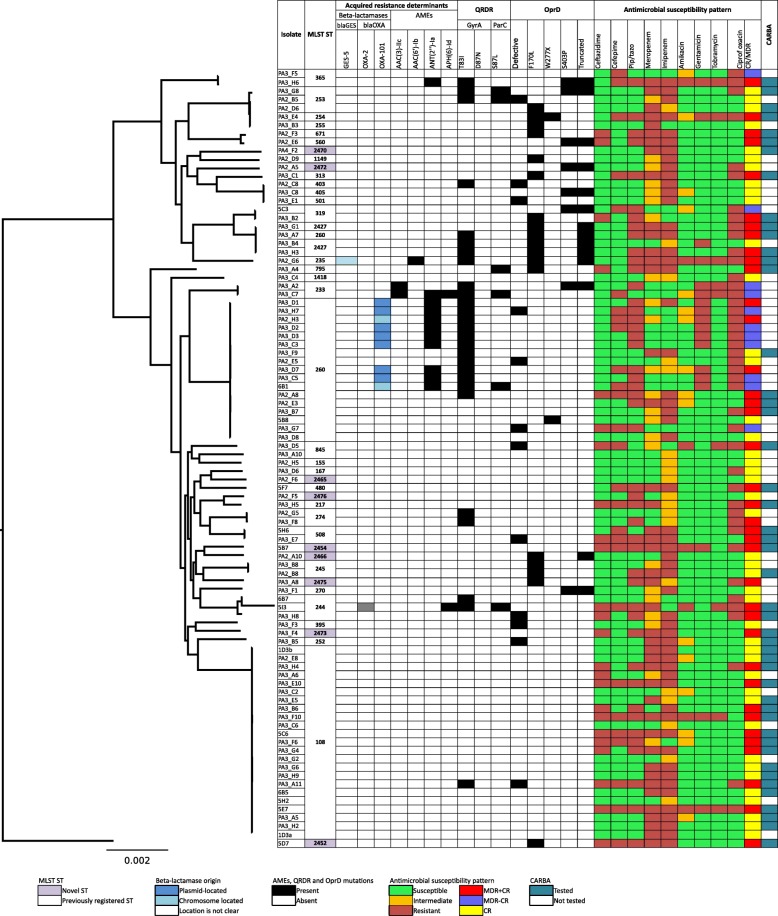
Analysis of 92 sequenced carbapenem or/and multiresistant *P.aeruginosa* genomes. A maximum-likelihood tree and MLST analysis, presence of beta-lactamases and their location (either in plasmid or chromosome) and aminoglycoside modifying enzymes, selected mutations in quinolone resistance-determining region (QRDR) and OprD. Tested antimicrobial susceptibilities are presented as follows: green color – susceptible; orange color – intermediate and red – resistant strain. CARBA represents coordinated results of phenotypic testing of class B beta-lactamases by combined-disk method and PCR where tested strains are marked with a dark blue color

CR was detected in 81 (88%) strains but no production of KPC or MBL was found by using combined-disk method and PCR in 47 randomly selected CR strains (Fig. 1).

### Genetic relationship and spread

Ninety-two CR/MDR-PA strains were assigned to 36 different sequence types (Fig. 1), of which 9 strains were novel to the MLST database.

The most prevalent sequence types were ST108 (*n* = 23; 25%) and ST260 (*n* = 18; 20%). ST108 strains were found throughout the study, indicating an endemic spread in 6 different wards (mostly in 2 ICUs) of one hospital. Eleven patients carrying ST108 had nosocomial infection, mostly ventilator-associated pneumonia (10 cases). All ST108 strains were CR (22 resistant to imipenem and 18 to meropenem) and 9 strains were MDR.

ST260 was found in all participating hospitals, but almost half of the strains (*n* = 7; 39%) belonging to this ST were isolated from a single hospital that was linked to one ICU. In most of cases (*n* = 14; 78%) ST260 caused infection and was phenotypically tested as MDR.

Only one strain belonged to the so-called high-risk international clone (ST235).

### Resistance genes

#### Beta-lactamases

Clinically relevant beta-lactamases (OXA-101, OXA-2 and GES-5) were found in 12% of the strains. Oxacillinases belonging to class D were found in 10 isolates, with OXA-101 (*n* = 9; 10%) being the most frequent and isolated only in ST260. No class B beta-lactamases (IMP, VIM; SPM; GIM, NDM, FIM) were present. None of the strains produced carbapenemases.

Plasmid encoded β-lactamases were present in 3 (3%) of the strains. Only one plasmid encoded class A beta-lactamase, GES-5; an isolate that belongs to the previously well-known high-risk, clone ST235.

#### Aminoglycoside modifying enzymes (AME) and 16S rRNA methyltransferases

Clinically relevant AME genes were found in 14 (15%) strains, the most frequent being ANT(2″)-Ia, present in 11 (12%) strains, 9 of which belonged to ST260.

The AAC(6′)-Ib-cr cassette mediating resistance to both aminoglycosides and fluoroquinolones was found in one isolate belonging to ST235 phenotypically resistant to ciprofloxacin and amikacin, but sensitive to gentamicin and tobramycin.

No 16S rRNA methyltransferase coding genes were found.

#### Mutational resistance mechanisms

The *oprD* gene was defective in 12 strains (13%; 8 resistant to both imipenem and meropenem; 1 imipenem and 1 meropenem resistant, and 2 with carbapenem-susceptible) and mutated in 77 strains (84%). We found 3 previously described mutations triggering CR – F170 L (*n* = 18; 20%), S403P (*n* = 8; 9%) and W277X (*n* = 2; 2%). Statistical analysis showed an association between defective *oprD* and non-susceptibility to meropenem (9 meropenem resistant vs 3 meropenem sensitive strains; *p* < 0.05).

Having researched fluoroquinolone resistance-associated mutations in topoisomerase genes *gyrA (*T83I, D87N) and *parC* (S87 L), we found T83I mutation was present in 27 (29%) strains, of which 23 were ciprofloxacin resistant. D87N was found in one and *parC* S87 L substitution in 6 ciprofloxacin resistant strains. Both gyrA T83I and parC S87 L mutations correlated well with ciprofloxacin resistance (23 resistant vs 4 sensitive strains; *p* < 0.0001, and 4 resistant vs 2 sensitive strains; *p* < 0.01, respectively).

## Discussion

This is the first study that addresses clinical risk factors alongside with the presence of resistance mechanisms in a mixed population of hospitalized patients infected or colonized with CR/MDR-PA and covers all major Estonian hospitals. The observations show that: (1) the vast majority of affected patients were elderly and had a history of previous contact with healthcare institutions and/or multiple co-morbidities confirming results of previous studies [24]; (2) the 2 predominant STs (ST108 and ST260) we described had good spreading potential and have rarely been recorded in previous studies, suggesting high ST variability within PA and minimal entry of internationally spreading strains into Estonian hospitals; (3) most of the CR strains lacked clinically relevant beta-lactamases including carbapenemases and metallo-beta-lactamases, suggesting that CR is encoded by a selection of mutations in chromosomal genes; (4) correlation between *gyrA* T83I and *parC* S83 L mutations and quinolone resistance were in support of previous studies [25–27].

Considering the high rate of patients with advanced age, the high rate of contact with healthcare facilities, and association with multiple courses of prior antibiotic therapy, it is not unsurprising that they have been previously described as risk factors for PA infection [28]. However, none of these patients belonged to typical risk groups, such as burns, cystic fibrosis or febrile neutropenia, but more commonly had congestive cardiac insufficiency. Similarly, other studies have shown decreasing trends of PA in burn and oncologic patients in Europe and North-America [29]. The reason for these trends is not entirely clear, but the fact that most of the empirical treatment regimens include anti-pseudomonal antibiotics may be a contributing factor.

The high rate of patients with congestive heart disease is more difficult to explain. It may be a concomitant finding and not directly related to the colonization of CR/MDR-PA. This also highlights the fact that PA infection is not a disease strictly associated with people that have severe disturbances of immune or barrier systems, because it can also affect patients without obvious immune defects, such as the elderly.

We found that the pattern of antibiotic resistance is largely driven but 2 STs (ST108 and ST265) causing hospital-acquired infections (mainly ventilator-associated pneumonia) and at least 2 outbreaks. Neither of these strains belonged to well-known internationally spreading clones [7]. ST108 was mostly associated with a singularly CR phenotype and spread only in one hospital. On the other hand, ST260 strains were much more diffused in hospitals. These strains usually had multiresistant phenotypes and carried some resistance machinery (ANT(2″)-Ia, OXA-101 and GyrA T83I mutation). Hence ST may have the potential for becoming a new high-risk clone. ST260 has been previously described in different human settings and in different geographical regions; it is mostly associated with MDR or XDR phenotype, but never with outbreaks [30–33].

CR of PA, reaching up to 20.4% has been a problem within Estonian hospitals for years [34]; however, potential resistance mechanisms have remained unidentified [11], and unfortunately we do not have definite answers from this study. In contrast to other European studies in which the most important trigger of resistance is the production of horizontally-acquired beta-lactamases (mainly belonging to Ambler class B), we found none of these strains, only a few clinically relevant beta-lactamases, of which only one (GES-5) has been described as having carbapenemase activity [20]. We detected a correlation between defective *oprD* and meropenem-resistance in 10 strains and a single mutation previously associated with carbapenem resistance in 25 strains [27]. These mechanisms, however, did not explain resistant phenotype of the remaining 46 strains. Probably a cascade of mutations that were not addressed in this study, including structural modifications in AmpC, peptidoglycan recycling genes and mutations leading to efflux pump overexpression, are required to facilitate phenotypic resistance.

Our data indicates the importance of 2 locally spread resistant clones in spite of low antibiotic consumption; tackling the main spreading routes of these clones could significantly reduce the burden of CR/MDR-PA. Because both the spreading clones had been isolated from respiratory secretions and associated with mechanical ventilation or bronchoscopy, the procedures related to the maintenance of the airway should be the focal point in the prevention of colonization and infections with CR/MDR-PA. Updating both the knowledge and skills of basic hand hygiene and isolation methods, improving oral hygiene practices, and revising bronchoscope cleaning techniques, including disinfection processes, can be effective in aborting PA outbreaks [35]. Infection control measures could be significantly improved by implementing high resolution molecular identification techniques (egg. WGS, MLVA) into everyday practice for rapid detection of outbreaks and stopping the spread of multiresistant microorganism and thus should be encouraged [36].

Some limitations of our study should be noted. Because samples were identified by microbiology laboratories and taken on discretion of treating physicians, there is a possibility that some patients, especially asymptomatic carriers, were not sampled. This leaves a gap in our understanding of how MDR-PA circulates in Estonian hospitals. Secondly, E-test was used for susceptibility measurement instead of microdilution that is EUCAST suggested reference method. Still, E-test results have correlated well with MICs generated by the dilution methods [37] and thus we believe that our results are reliable. Despite these limitations we believe that our results allow us to draw adequate conclusions.

## Conclusions

PA is a pathogen that affects not only immunocompromised, but also elderly multi-morbid patients. It is characterized by wide genetic diversity and spread via local rather than global clones in Estonian hospitals. The resistance machinery of PA is complex with only few certain correlations between genotypic and phenotypic resistance. Many different genetic changes may be required to develop the resistance pattern observed in phenotypic tests. High resolution genotyping methods are very valuable for tracking the spread of outbreaks, and therefore it is crucial to encourage the use of sequence-based methods in everyday practice.

## Additional files

Additional file 1: **Table S1.** Characteristics of patients with hospital-acquired infections. (DOC 34 kb)

Additional file 2: **Table S2.** Antibiotic groups used in prior 90 days before resistant CR/MDR-PA isolation. (DOC 36 kb)

## Abbreviations

AME: Aminoglycoside modifying enzymes
CR: Carbapenem resistant
CR/MDR-PA: Carbapenem and/or multidrug resistant *P. aeruginosa*
ECDC: European Centre for Disease Prevention and Control
EUCAST: European Committee on Antimicrobial Susceptibility Testing
I: Intermediately susceptible
KPC: *K.pneumoniae* carbapenemase
MBL: Metallo-beta-lactamase
MDR: Multidrug-resistant
MDR-PA: Multidrug-resistant *Pseudomonas aeruginosa*
MIC: Minimal inhibitory concentration
MIC_50_: Minimal inhibitory concentration at 50%
MIC_90_: Minimal inhibitory concentration at 90%
MLST: Multi-locus sequence typing
PA: *Pseudomonas aeruginosa*
QRDR: Quinolone resistance-determining region
R: Resistant
S: Susceptible
ST: Sequence type
WGS: Whole genome sequencing
XDR: Extremely drug resistant
