# Supplementary material for: Multidrug resistant Pseudomonas aeruginosa in Estonian hospitals
Source: BMC Infect Dis. 2018 Oct 11;18:513. doi: 10.1186/s12879-018-3421-1 (PMC6182868; doi:10.1186/s12879-018-3421-1)
Supplement: Supplementary file 1 — Table S1. Characteristics of patients with hospital-acquired infections. (DOC 34 kb) [file 12879_2018_3421_MOESM1_ESM.doc]

**Additional file 1: Table S1.**

Characteristics of patients with hospital-acquired infections (n=44)

| **Demographics** | N (%) |
| --- | --- |
| Male | 31 (70.5) |
| Age: median; years (interquartile range) | 67 (54-74) |
| Patients ≥ 65 years (%) | 23 (52) |
|  | |
| **Clinical infections due to resistant *P. aeruginosa*** |  |
| Respiratory tract infection; ventilator-associated pneumonia | 28 (64%); 22 (50%) |
| Surgical site infection | 6 (14%) |
| Skin and soft tissue infection | 5 (11%) |
| Other | 5 (11%) |
|  | |
| Median overall LOS*, days (IQR) | 15.5 (9-30) |
| Proportion with a long LOS* (≥8 days) | 34 (77) |
| ICU stay (%); duration in days: median (IQR) | 30 (68%); 13.5 (7.75-25) |
|  | |

*LOS - length of hospital stay before resistant CR/MDR-PA isolation
