# Supplementary material for: Multidrug resistant Pseudomonas aeruginosa in Estonian hospitals
Source: BMC Infect Dis. 2018 Oct 11;18:513. doi: 10.1186/s12879-018-3421-1 (PMC6182868; doi:10.1186/s12879-018-3421-1)
Supplement: Supplementary file 2 — Table S2. Antibiotic groups used in prior 90 days before resistant CR/MDR-PA isolation. (DOC 36 kb) [file 12879_2018_3421_MOESM2_ESM.doc]

**Additional file 2: Table S2.**

*Antibiotic groups used in prior 90 days before resistant CR/MDR-PA isolation*

| Antibiotic treatment | N of patients (%) | Mean(±SD) duration in days |
| --- | --- | --- |
| Beta-lactam group | 79 (86) |  |
| Aminopenicillins | 3 (3) | 3±2.8 |
| Flucloxacillins | 7 (8) | 5±0.9 |
| Penicillin/beta-lactam inhibitor | 25 (27) | 11±8.5 |
| Cephalosporins  1^st^ –2^nd^ generation cephalosporins  3^rd^ generation cephalosporins  4^th^ generation cephalosporins | 38 (41)  12 (13)  16 (17) | 7.1±6.9  8.8±2.9  6.1±3.7 |
| Carbapenems | 40 (43) | 9.5±6.2 |
|  |  |  |
| Sulfonamides and trimethoprim | 8 (9) | 8.3±3.4* |
| Macrolides | 6 (7) | 3.7±2.4 |
| Lincosamides | 4 (4) | 10.3±7.2 |
| Aminoglycosides | 11 (12) | 5.3±2.9 |
| Fluoroquinolones | 14 (15) | 7.3±4.6** |
| Glycopeptides | 9 (10) | 8.8±5.4 |
| Polymyxins | 4 (4) | 10.8±8.5 |
| Metronidazole | 13 (14) | 6.6±4.3 |
| Other | 3 (3) | 10±5.6 |
|  |  |  |

*A patient receiving TMP/SMX prophylactically 480 mg every other day for 60 days was removed from calculation

**A patient receiving ciprofloxacin for 47 days was removed from calculation
